# High malaria parasitemia in an Italian man and severe delayed hemolytic anemia

**DOI:** 10.1002/ccr3.9292

**Published:** 2024-11-07

**Authors:** Massimiliano Lanzafame, Giovanni Mori, Emanuela Lattuada, Claudio Scarparo

**Affiliations:** ^1^ Unit of Infectious Diseases, Santa Chiara Hospital, Azienda Provinciale per i Servizi Sanitari (APSS) Trento Italy; ^2^ Unit of Microbiology, Santa Chiara Hospital, Azienda Provinciale per i Servizi Sanitari (APSS) Trento Italy

**Keywords:** anemia, artesunate treatment, high parasitemia, Severe malaria

## Abstract

Parenteral artesunate has become the choice treatment of cases of high parasitemia malaria. We report the case of an Italian man with a high malaria parasitemia who promptly improved with intravenous artenusate, but who developed a delayed severe hemolytic anemia associated with intravenous artesunate treatment, requiring blood trasfusions.

## 1 OBJECTIVES

Parenteral artesunate is the choice treatment of cases of high parasitemia malaria. Compared with quinine, artemisinin reduces faster the level of parasitemia. Artemisin derivatives are rapidly parasiticidal,^1^ explaining their advantage in the treatment of high malaria parasitemia. However parenteral artesunate therapy is linked to development of delayed hemolytic anemia requiring blood trasfusions.^2^ Here we report the case of an italian man with a high malaria parasitemia who promptly improved with intravenous artenusate, but who developed a delayed severe hemolytic anemia associated with intravenous artesunate treatment, requiring blood trasfusions.

## 2 CASE DESCRIPTION

A 62‐year‐old Italian man, at begining of Febraury 2024, came back from Uganda after a 4 week stay. The same day of the return he developed fever, headhache, and diarrhea. He did not take malaria prophylaxis. On admission he was feverish (37.8°C) alert, conscious and with a normal mental status. His blood pressure was 103/59 mmHg, heart rate of 103 rhythmic, and a oxyhaemoglobin saturation of 97%.In emergency department (03 Febraury) his hemoglobin level was 15.3 g/dL (n.v. 13.5–18 g/dL) and the platelet count was 15,000/mm^3^ (n.v.150, 000‐400, 00/mm^3^).Protein C reactive was 140 mg/L (n.v. < 6 mg/L), creatinine level 1.42 mg/dL (n.v.0.67–1.17 mg/dL), lactate dehydrogenase was 875 U/L (n.v. 87–241 U/L), D‐dimer 88, 777 μg/mL (n.v. 0–0.057 μg/mL), total bilirubin level was 6.2 mg/dL (n.v.0–1.2 mg/dL) and procalcitonin was 21.60 ng/mL (n.v.0.50 ng/mL). The patient did not diagnosed with disseminated intravascular coagulation and he did not experience any bleeding symptoms. The day after (04 Febraury) hemoglobin level dropped to 11.4 g/dL.

**FIGURE 1:**
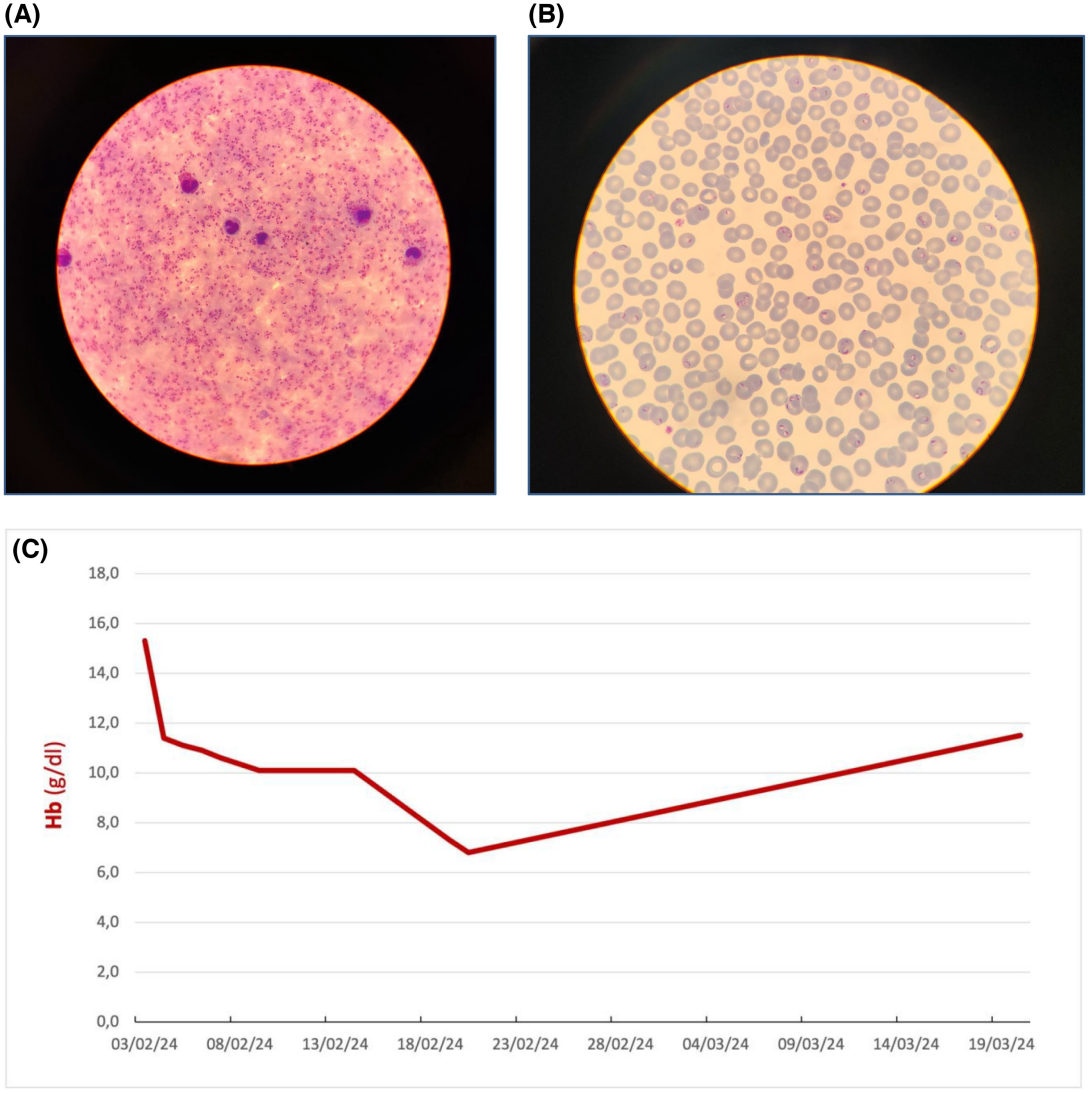
(A) High infestation with plasmodium falciparum with a parasitemia density of 25% as showed in the thick drop. (B) High infestation with Plasmodium falciparum with a parasitemia density of 25% as showed in the thin smear. (C) Trend of hemoglobin level.

Blood samples showed very high infestation with Plasmodium falciparum with a parasitemia density of 25% as showed in the thick drop (Figure 1A) and thin smear (Figure 1B).The patient was treated with intravenous artenusate for 3 days and then with oral combination of piperaquine tetraphosphate and artenimol for other 3 days. On 5 Febraury hemoglobin level was 11.1 g/dL.

Four days later parasitemia index was <0.01%. On 7 Febraury it was 10.6 g/dL and 7 days after was discharged in good clinical conditions with an hemoglobin value of 10.1 g/dL, platelet count of 199, 000/mm3,lactate dehydrogenase of 332 U/L and an aptoglobin value <0.078 g/L (n.v. 0.4–2 g/L) and 0.9% of reticulocytes (n.v.0.6–2.1).

Five days after discharge (19 Febraury) hemoglobin value was 7.3 g/L, total bilirubin level 3.1 mg/dL, gamma‐GT 277 U/L (10–80 U/L), lactate dehydrogenase of 1454 U/L and aptoglobin value was <0.078 g/L.The day after (20 Febraury) hemoglobin dropped to 6.8 g/dL and he necessitated blood transfusions (3 Units). One month later (19 March) he felt well with an hemoglobin value of 11.5 g/dL.In the graphic his trend of hemoglobin is showed in the graphic (Figure 1C).The treatment of severe malaria requires prompt, safe, and effective intravenous antimalarials. Many oral and intravenous agents are available worldwide for the treatment of malaria. A Cochrane Database Systematic Review, published in 2012, clearly showed the superiority of parenteral artesunate over quinine for the treatment of severe malaria.^3^ Studies conducted in travelers reported a good overall tolerance despite dealyed haemolytic anemia episodies has been showed to be associated to parenteneral use of artesunate.^2^

Clinicians must be aware of this possible complication and they must remember to check hemoglobin level also after symptoms resolution and hospital discharge of the patients.

## AUTHOR CONTRIBUTIONS

**Massimiliano Lanzafame:** Conceptualization; writing – original draft. **Giovanni Mori:** Writing – review and editing. **Emanuela Lattuada:** Investigation; validation. **Claudio Scarparo:** Formal analysis; resources.

## FUNDING INFORMATION

None.

## CONFLICT OF INTEREST STATEMENT

The authors declare that they have no conflict of interest.

## CONSENT

The patient provided a written consent for the use of his clinical data for scientific purposes.

## Data Availability

All data underlying the results are available as part of the article, and no additional source data is required.
